# Splice-specific deficiency of the PTSD-associated gene PAC1 leads to a paradoxical age-dependent stress behavior

**DOI:** 10.1038/s41598-020-66447-2

**Published:** 2020-06-12

**Authors:** Jakob Biran, Michael Gliksberg, Ido Shirat, Amrutha Swaminathan, Talia Levitas-Djerbi, Lior Appelbaum, Gil Levkowitz

**Affiliations:** 1 Department of Poultry and Aquaculture, Agricultural Research Organization, Rishon, Letziyon 7528809 Israel; 2 Department of Molecular Cell Biology, Weizmann Institute of Science, PO Box 26, Rehovot, 7610001 Israel; 3 The Faculty of Life Sciences and the Multidisciplinary Brain Research Center, Bar-Ilan University, Ramat-Gan, 5290002 Israel

**Keywords:** Stress and resilience, Behavioural genetics

## Abstract

The pituitary adenylate cyclase-activating polypeptide receptor (PAC1, also known as ADCYAP1R1) is associated with post-traumatic stress disorder and modulation of stress response in general. Alternative splicing of PAC1 results in multiple gene products, which differ in their mode of signalling and tissue distribution. However, the roles of distinct splice variants in the regulation of stress behavior is poorly understood. Alternative splicing of a short exon, which is known as the “hop cassette”, occurs during brain development and in response to stressful challenges. To examine the function of this variant, we generated a splice-specific zebrafish mutant lacking the hop cassette, which we designated ‘*hopless’*. We show that *hopless* mutant larvae display increased anxiety-like behavior, including reduced dark exploration and impaired habituation to dark exposure. Conversely, adult *hopless* mutants displayed superior ability to rebound from an acute stressor, as they exhibited reduced anxiety-like responses to an ensuing novelty stress. We propose that the developmental loss of a specific PAC1 splice variant mimics prolonged mild stress exposure, which in the long term, predisposes the organism’s stress response towards a resilient phenotype. Our study presents a unique genetic model demonstrating how early-life state of anxiety paradoxically correlates with reduced stress susceptibility in adulthood.

## Introduction

PAC1 (a.k.a. Adcyap1r1) is a G-protein coupled receptor (GPCR) that serves as the high-affinity receptor for the pituitary adenylate cyclase-activating polypeptide (PACAP). PAC1 has pleiotropic functions and was demonstrated to be involved in the regulation of several homeostatic processes including metabolic rate and food consumption^1,2^, circadian rhythm^3^ and, in particular, stress response^4,5^. Intracerebroventricular injection of PACAP increased phosphorylated cyclic AMP response element binding protein (pCREB) and corticotropin-releasing hormone (CRH) immunoreactivity in the rat paraventricular nucleus^6^. PACAP knockout mice display blunted hypothalamic CRH levels in response to restraint challenge^7^. PACAP/PAC1 signaling was also associated with hypothalamo-pituitary-adrenal activity and stress-related behaviors in humans and rodents^5,8,9^. Furthermore, this pathway was correlated with stress-related risky behaviors in human and rodents^10,11^. Overall, these findings support positive stress regulation by PAC1; yet, some reports suggest that it may also act to suppress stress phenotypes^4,12^.

It has been suggested that genetic vulnerability to post-traumatic stress disorder (PTSD) may depend on PAC1 expression and single-nucleotide polymorphism (SNP) in the PAC1 gene. Ressler *et al*. demonstrated that a specific PAC1 genotype is strongly correlated with susceptibility to PTSD in women, probably due to perturbed expression of PAC1 resulting in impaired stress responses^13^. The same PAC1 SNP was associated with PTSD in African-American females, emotional numbing in traumatized earthquake Chinese survivors, dark-enhanced startle response in children^14–16^, and with impaired hippocampal and amygdalar activation in response to fearful stimuli and contextual fear conditioning in non-traumatized individuals^17,18^. Moreover, PTSD was correlated with altered expression of PAC1 in the human cortex^13^.

These accumulating data have associated PAC1 to PTSD based on SNPs in the PAC1 gene promoter, global PAC1 expression or both. However, PAC1 mRNA undergoes extensive alternative splicing, resulting in the generation of several protein isoforms that differ in their ligand affinity and signal transduction cascades^19–21^. There are at least 17 known PAC1 splice isoforms. The predominant brain isoforms are PAC1-hop, which contains an alternatively spliced short exon termed the “hop cassette”, and PAC1-short that lacks it^22–24^. The hop exon encodes 28 amino acids of the third intracellular loop of this GPCR. PAC1-hop is expressed in neuroendocrine cells in mammals and fish and it is widely expressed in the brain and gonad tissues^21,22,25^. The splicing of the hop cassette occurs during brain development and in response to stressful challenges^4,21^. However, the specific roles of distinct splice variants in the regulation of stress-related behaviors are yet to be uncovered.

Neuroendocrine systems mediating homeostatic response to stressful challenges are well conserved between mammals and fish; indeed, PAC1 splice variants have been identified in zebrafish^21,26^. The zebrafish genome contains two PAC1 paralogs, namely *pac1a* and *pac1b;* however, only the *pac1a* gene contains the hop exon. We have previously demonstrated that alternative splicing of PAC1 is induced by an acute stress challenge to mice, which suggested that the ratio between PAC1-hop and PAC1-short isoforms may be involved in the adaptive response to stress^4^. We further demonstrated that zebrafish larvae injected with an antisense oligonucleotide that prevents the inclusion of the hop cassette displayed prolonged increase of *crh* transcription and impaired anxiety-like dark avoidance behavior^4^. In the present study, we examined the short- and long-term behavioral consequences of this splice-specific PAC1 deficiency by generating a germline-transmitted zebrafish mutant lacking the hop cassette, which we designated ‘*hopless’*. We show that while *hopless* larvae display increased anxiety-like and stress related behaviors, adult *hopless* mutants exhibit increased resilience to acute stress. These findings suggest that PAC1-hop splice isoform is involved in the developmental establishment of stress responsiveness.

## Results

### Generation of zebrafish mutant with germline deletion of the hop-coding exon

**Figure 1:**
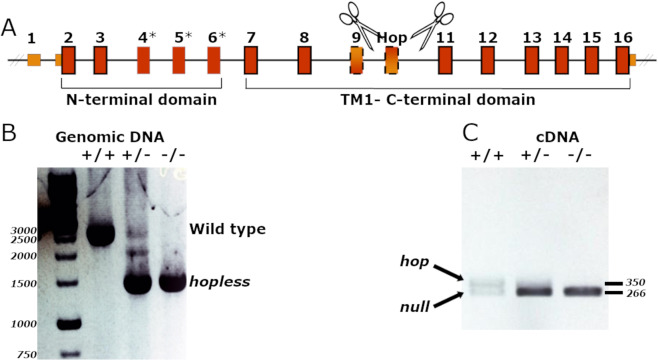
Establishment of *hopless*^*wz18*^ zebrafish mutant. (**A**) A pair of gRNAs (indicated by scissors) were designed to target the upstream and downstream genomic regions flanking the zebrafish hop cassette. (**B**) PCR amplification of the genomic hop-cassette site demonstrate a single truncated product in the mutant genome. (**C**) Amplification of PAC1-hop region in the zebrafish PAC1 mRNA shows loss of the hop isoform in cDNA produced from 6 dpf larvae. Plus sign represents wild-type allele; minus sign indicates *hopless* allele. Full length source electorophoresis gel images are provided in Supplementary Fig. 1.

As mentioned, while PAC1 is known to be involved in modulation of stress response, the functions of each of its many splice variants are poorly understood. To examine the specific role of PAC1-hop splice variant, we have generated a zebrafish mutant that harbors a deletion of the alternatively-spliced hop exon of *pac1a* gene (Fig. 1A). To generate this mutant, we used the CRISPR/Cas9 system to simultaneously target two intragenic sites around exon 10 of *pac1a* (Fig. 1A). To avoid introduction of a phantom exon due to partial intronic retention, gRNA binding sites were designed at least 600 bp upstream and downstream to the hop cassette, thus including its predicted cis-regulatory splice sites. Mosaic F_0_ fish identified for carrying large genomic deletions were outbred with *Tüpfel long fin* (*TL*) wild-type (WT) strain. Their offspring were screened for individuals carrying at least 1,600 bp deletion in their genomic DNA. Four individual F_1_ fish were identified as carriers of the same genomic ~1,600 bp indel. The resulting germline-transmitting mutant allele lacked the *hop* exon (Fig. 1B and Supp. Fig. 1A) as did the *pac1a* mRNA gene product (Fig. 1C and Supp. Figure 1B); hence, we named this mutant ‘*hopless*’. Sequencing of *pac1a* cDNA that was derived from the *hopless* mutant line confirmed the in-frame deletion of exon 10 in the mature mRNA gene product, without perturbing the amino acid reading frame of the flanking exons 9 and 11.

Finally, homozygous *hopless* fish were viable and fertile and displayed no detectable morphological abnormalities compared to their WT siblings. Therefore, the generated mutant allowed us to assess the behavioral effects of the deleted *hop* exon in both larval and adult stages.

### *hopless* mutant larvae display heightened anxiety-like behaviors

**Figure 2:**
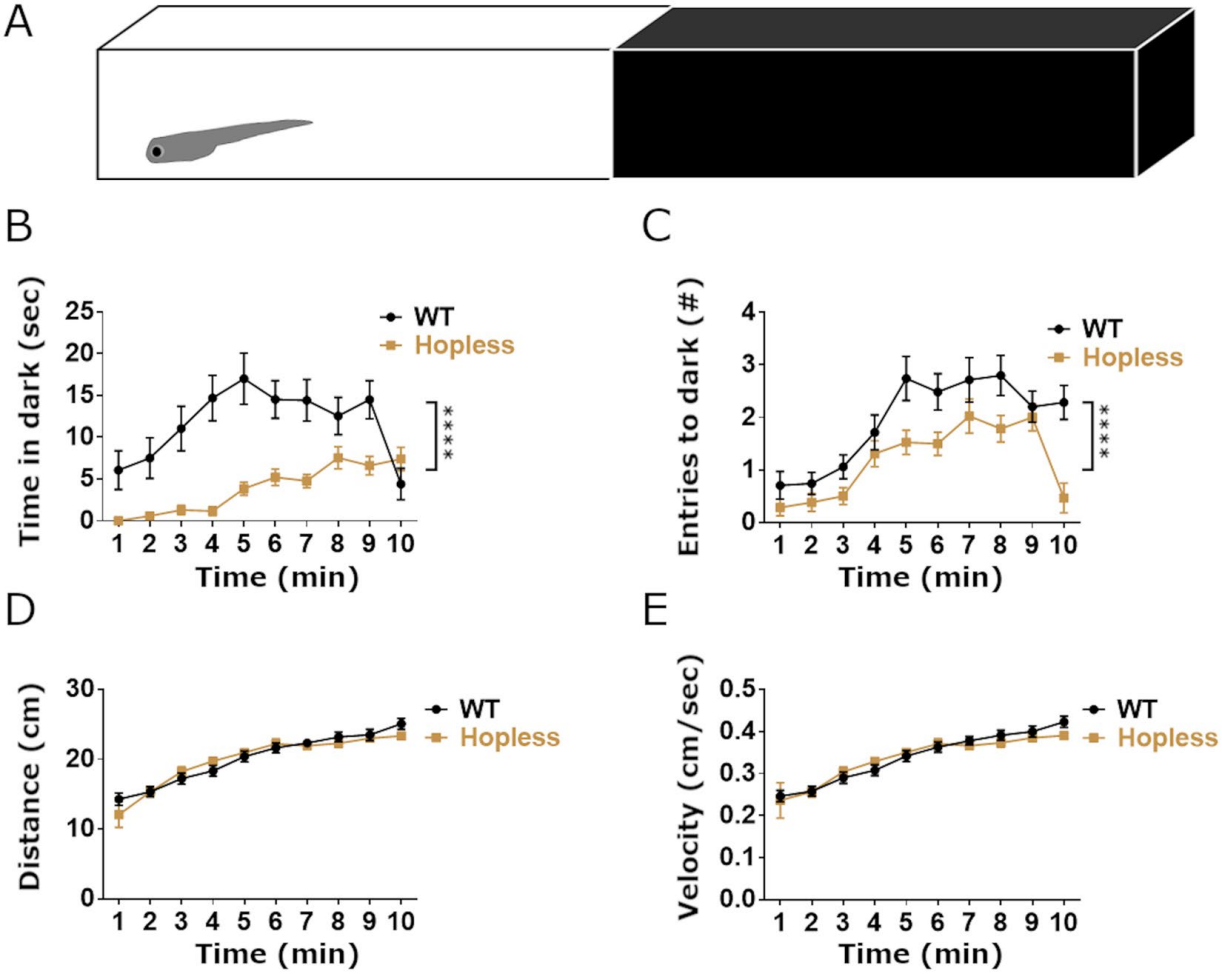
*Hopless* larvae display increased light preference in light-dark preference test. (**A**) Larvae were placed in the lit side of a half lit-half dark rectangular arena and their behavior was recorded for 10 minutes. Analysis of locomotor activity showed that *hopless* mutants spent significantly less time in the dark zone (**B**) and exhibited less entries into the dark (**C**) relative to WT larvae. Nonetheless, 6 dpf *hopless* larvae (n = 42) did not differ from WT controls (n = 35) in total distance swum (**D**) or average velocity (**E**). Data are presented as mean ± SEM of 1 minute time bins. Data were analyzed using 2-way ANOVA. For all analyzed parameters, a significant effect of time (p < 0.0001) was observed. Quadruple asterisks indicate significant difference between genotypes (p < 0.0001). No significant interaction was found for time*genotype.

To measure the behavioral effect of *hop* exon deficiency, we first used the light-dark preference test (Fig. 2A). In this assay, a larvae that is placed in a half lit, half dark arena displays a strong preference to the lit region^4,27^. The time spent in the dark is increased following exposure to anxiolytic drugs and, therefore, this behavioral assay is considered a measure of larval anxiety-like state^4,27^. We performed the test on 6 days post-fertilization (dpf) *hopless* larvae and genetically matched WT larvae. As previously demonstrated for larvae exposed to the anxiogenic caffeine^27^, as well as by our previous report using interference with PAC1a-hop alternative splicing with antisense morpholino oligonucleotide^4^, we found that *hopless* larvae generated by permanent genomic editing by means of CRISPR-Cas9, displayed significant reduction in the time spent in the dark zone, as well as in the number of entries to the dark zone (Fig. 2B,C). The observed phenotype was not due to locomotion defects, as the distance and speed of swimming of *hopless* larvae was similar to those of WT larvae throughout the test (Fig. 2D,E).

**Figure 3:**
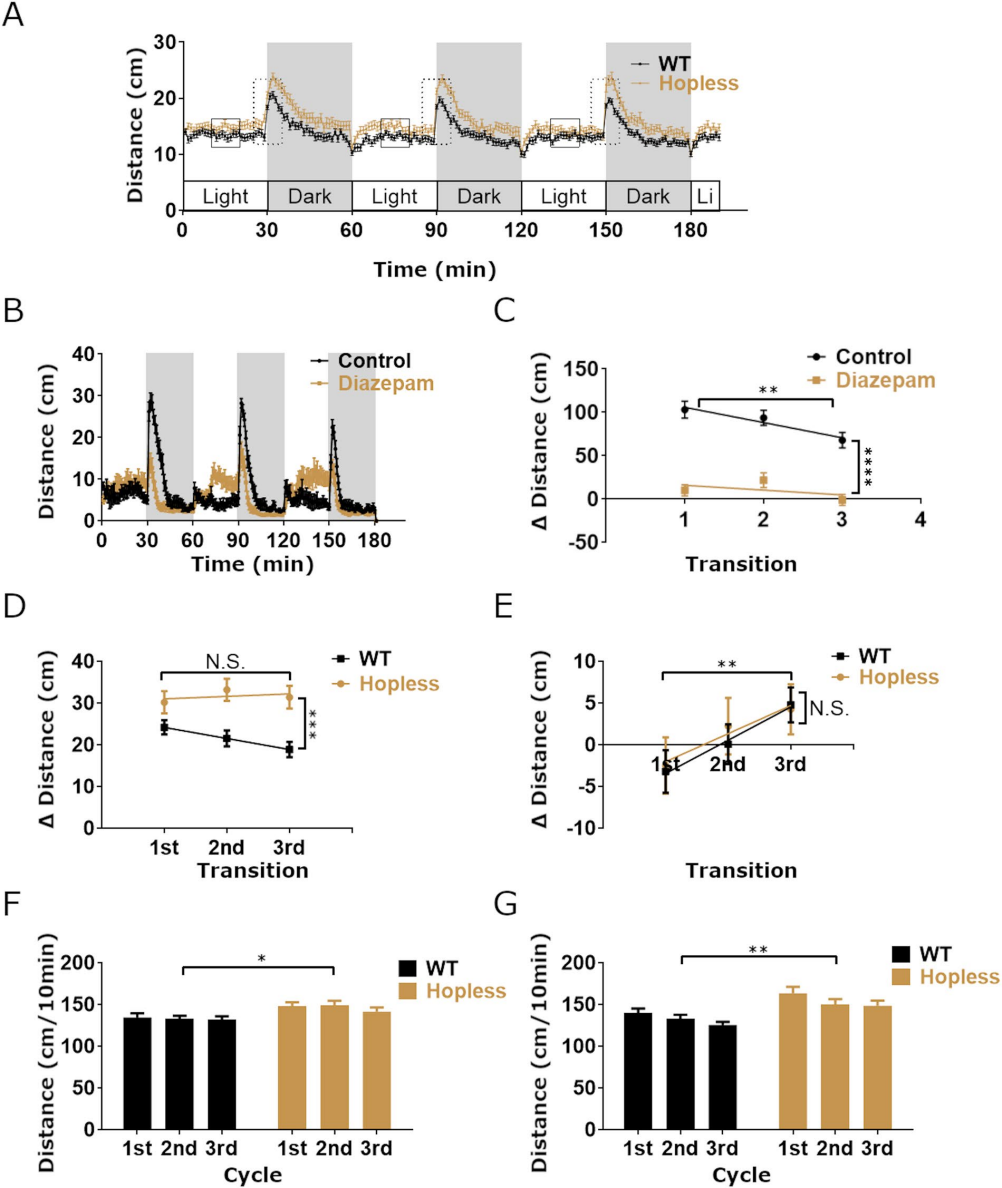
*Hopless* larvae display impaired stress-related behavior in light-dark transitions assay. Mean total locomotor activity of 6 dpf wild type (WT; n = 48) and *hopless* mutant (n = 41) larvae was measured during 3 h of 30 min alternating light-dark cycles (**A**). Mean total activity of each genotype was calculated for the middle 10 min interval of each phase (black squares in **A**) or as the difference in total activity between 5 min post-transition and 5 min pre-transition (dashed rectangles in **A**). WT larvae (n = 24) that were treated with 5 µM diazepam displayed similar activity pattern to non-treated siblings (n = 24; **B**); yet, treated larvae displayed reduced dark-enhanced activity (**C**). Analysis of larval activity during light-to-dark transitions, calculated as the delta of the total distance swum 5 min post-transition to 5 min pre-transition, revealed that WT larvae gradually decreased their dark-enhanced activity with each cycle, representing habituation to the challenge, whereas *hopless* larvae failed to display such habituation (**D**). During the opposite dark-to-light transitions no differential responses between genotypes were observed (**E**). In general, *hopless* larvae exhibited significantly increased activity under both light and dark conditions (**F** and **G**, respectively). Data were analyzed using 2-way ANOVA. Single asterisks indicate p < 0.05; double asterisks indicate p < 0.01; triple asterisks indicate p < 0.001; quadruple asterisks indicate p < 0.0001. No significant interaction was found for time*genotype.

We next performed the light-dark transition assay, which measures the locomotion response of larvae to a sequence of sudden transitions between bright light and total darkness. Larvae tend to respond to sudden darkness by a short burst of vigorous swimming, the intensity and duration of which are increased under stressful conditions^28^. We analyzed larval locomotion using 30 minute dark/light alternating periods^29^, as we found that this interval enables complete rebound from the stressful transitions (Fig. 3A). Results showed that immediately after light-to-dark transitions, WT larvae displayed darting behavior followed by a gradual decrease in locomotion, presumably due to acclimatization to the darkened environment (Fig. 3A). Conversely, dark-to-light transitions induced a short freezing episode, which was followed by a rebound to normal locomotion (Fig. 3A). Moreover, the intensity of larval locomotion response to the above transitions was reduced during repeated light-dark cycles, indicating that they habituated to the sudden changes in the environment. To test whether the light-to-dark transition measures anxiety-like response, WT larvae were treated with diazepam, which was shown to be anxiolytic in both larvae and adult zebrafish^27,30^. Diazepam-treated larvae displayed reduced darting activity during the transition from light to dark, suggesting that light-to-dark sensory challenge is anxiogenic (Fig. 3B,C).

In contrast to the behavioral pattern observed in WT fish, *hopless* larvae failed to habituate to repeated light-to-dark transitions, as their darting response did not change between the first and third transitions (Fig. 3A,D). Interestingly, their habituation to sudden dark-to-light transitions was similar to that of the control, suggesting that this challenge may be less stressful to the larvae (Fig. 3A,E). Finally, we observed that *hopless* larvae exhibited heightened locomotion in both light and dark phases. This may indicate an impaired response of this mutant to the novel environment of the behavioral arena (Fig. 3F,G).

Taken together, our results indicate that *hopless* mutant larvae, which lack the alternatively spliced *hop* exon, display heightened anxiety-like responses to sensory-based light-dark stress stimuli.

### Adult *hopless* zebrafish display impaired behavioral response to novel environment

To assess whether the stress-related behavioral phenotypes of the *hopless* mutant persist to adulthood, we utilized the novel tank diving and open field assays. When adult zebrafish are introduced to a novel tank, they initially dive to the bottom half of the tank and, over time, they gradually begin to explore its top half ^31^. This stereotypical behavior is commonly used to measure stress and anxiety-like response of zebrafish to a novel environment^32,33^. When we introduced adult WT or *hopless* zebrafish to the novel tank arena with no prior stressful challenge, both genotypes displayed similar behaviors, as reflected by similar swimming distance and velocity (Supp. Figure 2A,B), as well as by the similar number of entries and time spent in the top half of the tank (Supp. Figure 2C,D). Likewise, no significant differences between WT and *hopless* zebrafish were found in open field behavioral parameters, including locomotion, place preference towards center versus the walls, and freezing, which are also considered anxiety-like responses (Supp. Figure 3). These results indicate that naïve adult *hopless* mutants do not display differential response in novelty-based behavioral assays.

We next examined the behavioral responses of *hopless* adults in novelty-based assays following an acute stressful challenge, namely a short restraint in a confined space. Wild-type and *hopless* siblings were restrained for 2 minutes in a 50 ml conical tube containing 5 ml of water, such that the fish was covered with water allowing normal breathing, but in a highly confined space. Thereafter, both genotypes were analyzed in the novel tank diving and open field assays. Novel tank diving assay duration was extended to 14 minutes, in order to allow the restrained fish to recover from the stressful restraint challenge.

**Figure 4:**
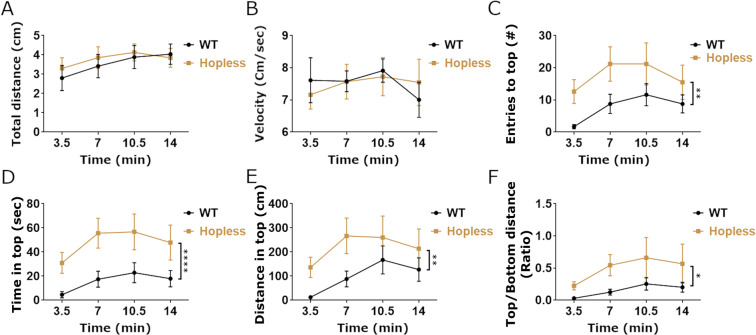
A preceding stressful challenge leads to blunted stress-related behavior of *hopless* adults in the novel tank assay. The behavioral response of adult *hopless* mutants (n = 11) and WT siblings (n = 13) to a novel tank was measured following 2 min restraint in a confined space. The two genotypes exhibited similar overall activity, as seen in their mean swimming distance and velocity (**A**,**B**). Nonetheless, *hopless* mutants entered more times to the top half of the tank, spent more time and covered more distance there, even when normalized to distance swum at the bottom of the tank (**C**–**F**). Data are presented as mean ± SEM of 3.5 minute time bins. Data were analyzed using 2-way ANOVA. Single asterisks indicate p < 0.05; double asterisks indicate p < 0.01; quadruple asterisks indicate p < 0.0001.

**Figure 5:**
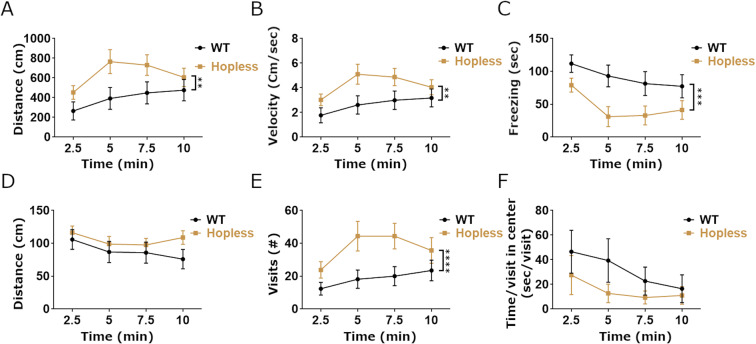
A preceding stressful challenge leads to blunted stress-related behavior of adult *hopless* zebrafish in the open field assay. Adult *hopless* mutants (n = 10) and WT siblings (n = 13) behavior was measured in a novel open field arena following 2 min restraint in a confined space. A significant reduction is seen in activity level of WT, but not of *hopless* mutant zebrafish (**A**,**B**). This effect was probably due to the increased freezing of WT adults (**C**). No significant difference was seen in the time spent in the center of the open field arena (**D**); however, *hopless* mutants displayed significantly increased number of visits to the arena center and a tendency (p = 0.09) to spend less time in each visit to center (**E**,**F**). Data are presented as mean ± SEM of 2.5 minute time bins. Data were analyzed using 2-way ANOVA. Double asterisks indicate p < 0.01; triple asterisks indicate p < 0.001; quadruple asterisks indicate p < 0.0001.

When placed in the novel tank arena, stressed fish of both genotypes covered the same distance with a similar swim velocity (Fig. 4A,B). However, *hopless* mutants displayed more entries to the top half of the tank, indicative of reduced stress behavior (Fig. 4C). *hopless* mutants also spent more time and covered more distance in the top half of the arena (Fig. 4D–F). When analyzed in the open field arena, stressed *hopless* mutants covered more distance with higher average velocity than stressed WT zebrafish (Fig. 5A,B). This effect was probably due to the reduced freezing of *hopless* mutants (Fig. 5C). Although no significant difference was found in the time spent in the center of the arena, *hopless* mutants performed significantly more visits to the center and showed a tendency to spend less time per visit, indicative of increased arena exploration (Fig. 5D–F). It is noteworthy that following restraint, WT fish froze also in the center of the arena (data not shown), which may explain, at least in part, the lack of difference in time spent in the center. We conclude that adult *hopless* mutants display improved behavioral recovery following an acute stress, suggesting that they are more resilient to stressful challenges.

## Discussion

PAC1 is an important player in the regulation of physiological and behavioral stress responses. Perturbation of its expression was associated with PTSD and impaired stress responses^34,35^. Yet, although PAC1 undergoes extensive alternative splicing, the specific roles of its isoforms in the regulation of stress response have not been elucidated. In the present study, we have generated a unique genetic model, which lacks the *hop* exon, and used this mutant to examine the role of PAC1-hop splice variant in the regulation of stress-related behaviors throughout life. Our results revealed an intriguing phenomenon of an age-dependent switch in stress-related behaviors. As larvae, *hopless* mutants display increased anxiety-like responses. Conversely, adult *hopless* fish rebounded better from a major stressor (restraint), displaying reduced anxiety-like responses to a subsequent milder stressor (introduction into novel environment), which indicates improved resilience to stress during adulthood. We suggest that the developmental loss of a specific PAC1 splice variant mimics a heightened anxiety state that, in the long-term, renders an improved ability to cope with stressful challenges.

PAC1 splice isoforms are known to mediate distinct signal transduction pathways due to differential coupling to G-proteins^21,36,37^. The PAC1-short isoform, which does not contain the hop exon, displays very low or no activation of PLCβ, whereas PAC1-hop1 switches its mode of signaling from AC to PLCβ^25,38–41^. PAC1-hop1 was also shown to regulate Ca^2+^ mobilization and neurosecretion^42–45^. Such differential signal transduction could affect behavioral phenotypes, as previously demonstrated for other GPCRs^46–49^. Accordingly, we have previously shown in adult mice that acute foot-shock stressor induces a change in the ratio between PAC1-hop and PAC1-short splice isoforms in the hypothalamic paraventricular nucleus^4^.

Using an antisense morpholino oligonucleotide in larval fish, we also showed that interference with PAC1a-hop alternative splicing leads to impaired *crh* transcriptional dynamics, as well as adaptation to a stressful challenge^4^. However, due to the transient nature of this gene-knockdown approach, the long-term consequences of developmental deficiency of *pac1*-*hop* on adult stress responses were not examined. In the present study, we utilized the CRISPR/Cas9 method to generate a germline mutation resulting in the loss of *pac1*-*hop* isoform mRNA without affecting the generation of the other *pac1* isoforms, allowing functional investigation of the role of *pac1*-*hop* throughout life.

Early life stress exposure may have negative or positive impact on adult psychological well-being. For example, in humans early life stress is known to increase the risk for psychopathologies in adulthood^50,51^. In particular, PTSD was recently correlated with childhood anxiety of both military and civilian men^51^. Nonetheless, more than 60 years ago, Levine demonstrated that mild stress exposure can lead to an inoculation effect in the adult rat, making it more resilient to stressors^52^. These findings were later validated in mice and squirrel monkeys^53,54^. In line with this notion, in our study *hopless* larvae exhibited increased anxiety-like behavior in the light-dark preference test and reduced habituation to repeated light-to-dark transitions. However, pre-challenged adult mutants displayed a blunted stress response of increased exploration and reduced freezing in both novel tank diving and open field assays, indicating enhanced resilience to novelty stressors.

As mentioned above, in various animal models, PAC1 signaling has been mainly associated with activation of physiological and behavioral stress responses, including PTSD^10,13,55–57^. Infusion of PACAP, the high-affinity PAC1 ligand, to the bed nucleus of stria terminalis potentiated anxiogenic response in pre-stressed but not in naïve male rats, which was likely conveyed by PAC1 signaling^9,58^. PAC1-KO mice display blunted elevation in corticosterone following acute restraint stress, but not under naïve conditions^5^. However, our current study highlights the need for better understanding of the physiological and psychological functions of specific PAC1 splice isoforms. Generation of additional site- and splice-specific genetic models is needed to further elucidate mechanisms underlying splicing-based regulation of the stress response.

Although the underlying mechanism for the switch in stress responsiveness of *hopless* mutants studied herein is still unknown, some studies may provide hints. A dynamic shift in the ratio of PAC1-hop and PAC1-short isoforms was shown to regulate pro- and anti-mitogenic activity of PACAP in the developing brain^59^. The ratio of PAC1-hop2 mRNA to other PAC1 variants in the ventromedial nucleus of the hypothalamus of female rats appears to be dynamic, depending on different steroid environments^60^. This study suggests that steroid-regulated changes in the expression of PAC1 splice variant in the hypothalamus contribute to the effect of PACAP on female receptivity^60,61^. We have previously suggested that alternative splicing of the hop cassette serves as an ON/OFF stress switch^21^, in line with our finding that stress-induced alternative splicing of PAC1-hop is involved in the termination of *crh* transcription and adaptive anxiety-like larval behavior. Larvae with depleted PAC1-hop1 display delayed dark-avoidance recovery following a stressful challenge response^4^.

Taken together, our findings suggest that PAC1-hop is involved in the developmental establishment of stress responsiveness. Thus, in our current study, *hopless* larvae likely experienced an impaired ability to terminate their stress response, which presumably led to prolonged and possibly heightened anxiety-like states during early life stages. These states could occur in response to any of the very mild stressful events that the animals experience during rearing in an enclosed laboratory setting, for example, the sudden daily switch from dark to light, encounter with human caretakers, tank cleaning, etc. We submit that in the case of the *hopless* mutant, this early life state of anxiety paradoxically correlates with reduced stress susceptibility in adulthood, reminiscent to the early-life stress inoculation proposed by Levin^52^ and others.

## Materials and Methods

### Animal care and maintenance

Zebrafish were bred and reared at 28.5 °C under 14 h/10 h light/dark cycle, according to standard protocols. Embryos were raised at 28.5 °C in 30% Danieau’s medium (0.17 mM NaCl, 0.21 mM KCl, 0.12 mM MgSO_4_, 0.18 mM Ca(NO_3_)_2_, 0.15 mM HEPES, pH 7.4) supplemented with 0.01 mg/L methylene blue. All experimental procedures were approved by and conducted in accordance with the Weizmann Institute’s Institutional Animal Care and Use Committee (IACUC).

### Genome editing using CRISPR/Cas9

To generate the *hopless*^*wz18*^ allele (ZFIN ID: ZDB-ALT-191028-3) we employed genome editing as previously described^62,63^, with slight modifications. Cas9 protein was produced by the Weizmann Institute of Science Protein Purification Unit using the pET-28b-Cas9-His (Alex Schier Lab Plasmids, Addgene, Cambridge, MA, United States) as a template. CRISPR sgRNAs (Supplementary Table S1) were designed using CHOPCHOP^64^. Oligonucleotide containing the SP6 promoter sequence upstream of specific target sites was annealed with a constant oligonucleotide bearing Cas9 binding site. sgRNA were generated by *in vitro* transcription using a SP6 RNA polymerase MEGA script SP6 kit (Life Technologies, United States) and purified using miRNeasy kit (Qiagen, Germantown, MD, United States). Cas9 protein (600 ng) and sgRNAs (1500–2000 ng) were co-injected to *Tupfel long fin* (TL) zygotes at the one-cell stage. PCR analysis of ten embryos was performed to evaluate genomic mutations and occurrence of large deletions induced by multiple gRNAs injection. Injected siblings were raised to adulthood and screened for large deletions in genomic-DNA (gDNA) extracted from tail clips. Fish that were found to be positive for large genomic deletions were crossed with TL zebrafish to identify germline transmission. Germline heterozygotes were genotyped using Sanger sequencing and fish carrying the *hopless* allele were crossed to generate homozygous mutants.

### DNA extraction and genotyping

gDNA was extracted from tail fin clips of adult fish or whole embryo. DNA was extracted according to^65^. Briefly, samples were digested at 50 °C with 1% SDS and proteinase K solution (20 mg/ml). Protein precipitation was performed by adding 5 M NaCl solution and centrifugation. Supernatant was transferred to a clean tube, followed by DNA precipitation using ice-cold isopropanol. DNA pellet was isolated by centrifugation and washed with 70% ethanol. DNA pellets were air-dried, dissolved in 50 µl double distilled water and stored at −20 °C until further analysis. The genomic region of *hop* cassette was amplified by using Taq DNA Polymerase Master Mix Red 2x reaction mix (Ampliqon, Odense, Denmark). Amplified DNA region was detected by 1–1.5% agarose gel electrophoresis, then extracted and purified by using the NucleoSpin Gel and PCR Clean-up (Macherey-Nagel, Düren, Germany). Purified DNA fragments were sequenced by the Biological Services Unit at the Weizmann Institute of Science. Results were analyzed by ApE-A software (version 2.0, by M. Wayne Davis, University of Utah). See Supplementary Table S1 for oligonucleotide sequences.

### Light/dark preference test

Anxiety-like behavior of zebrafish larvae was measured by recording the preference of 6-day-old zebrafish larvae for either side of a custom-made light-dark arena. The test was generally performed according to^4^ with slight modifications. Briefly, activity of individual larvae was recorded for a 10 min time period by high resolution infrared video camera (Flare 2M360-CL, I/O industries, London Ontario), with an image acquisition Sapera LT-development package (Teledyne Dalsa, Waterloo, Ontario) and recorded with Stream5 software (IO Industries, London, Ontario). The resulting videos were analyzed using EthoVision XT (Noldus Information Technology, Wageningen, Netherlands). *Hopless* larvae and matched TL control larvae were randomly assigned into separate arenas with bottom lighting. Each arena was divided into two equal compartments in which the top, bottom and sides of the dark half were covered with Optical Cast Infrared (IR) Longpass Filters, allowing penetration of infrared light (Edmund Optics, Barrington, NJ, USA). The total distance swum in the whole arena and in each compartment was calculated and compared for each genotype. Animals that stopped swimming for periods of 60 s or more were removed from the analysis.

### Larval light/dark transitions test

Larval responses to sudden transitions from light to dark and from dark to light were monitored using the Noldus DanioVision tracking system (Noldus Information Technology, Wageningen, Netherlands), as previously described^29^. For each experiment, individual larvae were placed in 48-well plates, which were put in the DanioVision system with light on for 1 h prior to the beginning of the trial. Larvae were subjected to three cycles of 30 min light/30 min dark followed by 10 min light. The experiment was repeated three times, each with a new batch of 6 dpf *hopless* and TL larvae. Total swimming distance and velocity were calculated using EthoVision XT Software (Noldus Information Technology). For the diazepam treatment experiment, larvae were exposed to 5 µM diazepam (Renaudin, France) for 2 h prior to the start of the behavioral testing. Control larvae were kept in similar conditions during this period. At the end of the incubation, larvae were transferred into individual wells of a 48-well plate and subjected to 30 min light/30 min dark cycles and analyzed as described above. The experiment was repeated four times, each with a new batch of 6 dpf TL larvae.

### Novel tank diving assay

Behavioral phenotyping was performed using a custom-made apparatus, as was previously described^66^. Novel tank diving assay was performed as was previously described^4^. Adult fish were individually placed in a rectangular arena (17.5 × 5 × 11.5 cm or 12 × 6.8 × 13 cm for naïve or stressed fish, respectively) and their swimming activity was recorded. Acquisition of 6 min videos for naïve and 14 min videos for stressed adult fish was performed using a 2M360-CL camera (IO Industries, London, Ontario), with an image acquisition Sapera LT-development package (Teledyne Dalsa, Waterloo, Ontario) and recorded with Stream5 software (IO Industries, London, Ontario). Tracking analysis was performed using EthoVision video tracking system (Noldus Information Technologies, Wageningen, Netherlands). In EthoVision, the arena was split in the middle into two zones (top and bottom) and fish activity was analyzed for the total distance moved, velocity, number of visits for each zone and for the whole arena. The experiment was repeated three times for naïve and three times for stressed fish (4–6 months old, total length ~2.5 cm, unsexed) originating from the same spawns of the behaviorally analyzed larvae. Fish were genotyped and housed in 3-L tanks according to their genotype (4–5 fish/L) for recovery at least one week prior to individual tracking of their behavior.

### Open field

Open field analysis was performed by placing individual naïve fish in a circular arena of 23 cm in diameter filled to a height of 5 cm or stressed fish placed in a rectangular arena (21 × 20 cm) filled to a height of 4 cm with regular system water. The arena was placed in a custom-built enclosure that restricted all visual cues from the laboratory frame, and was top-lit with ambient LED light. The assay was conducted within the same enclosure as the novel tank diving assay and using the same recording equipment. Swimming was recorded for 10 min for both naïve and stressed fish. In EthoVision, 85% of the arena was defined as “center” zone and positioned in the exact center of the arena. Fish activity was analyzed for the total distance moved, velocity, number of visits to center and their duration. The experiment was repeated three times for naïve and three times for stressed fish (4–6 months old, total length ~2.5 cm, unsexed) originating from the same spawns of the behaviorally analyzed larvae. Fish were genotyped and housed in 3-L tanks according to their genotype (4–5 fish/L) for recovery at least one week prior to individual tracking of their behavior.

### Restraint challenge

Previous reports of acute restraint stress demonstrated the use of 2 ml microtubes as physical restrainers for adult zebrafish inducing central and physiological stress responses^67,68^. Aiming to reduce possible physical strain and a milder anxiogenic effect, adult fish were individualy placed in a standard 50 ml conical tube with 5 ml of system water for 2 min. The tube was placed in an upright position such that the fish could not swim, but was completely covered with water to prevent dehydration or loss of oxygen. Following the 2 min restraint, the fish were introduced to the open field arena or novel tank diving arena for behavioral tracking.

### Statistical analysis

Data of a representative experiment for each paradigm are presented as mean ± standard error of the mean (SEM) and were analyzed using GraphPad Prism 7.01. The number of larvae or adult fish used for each experiment is indicated in figure legend. Replicate experiments were performed with similar animal quantities and resulted with similar phenotypes. All data sets were tested for departures from normality with Shapiro-Wilks test. Student’s *t*-test was used for all comparisons between two groups. 2-Way ANOVA was used for comparing multiple variables. Single asterisks indicates p < 0.05, double asterisks indicates <0.01, triple asterisks indicates p < 0.001 and quadruple asterisks indicates p < 0.0001.

## Supplementary information

Supplementary information.
