# Publisher Correction: Splice-specific deficiency of the PTSD-associated gene PAC1 leads to a paradoxical age-dependent stress behavior

**DOI:** 10.1038/s41598-020-69589-5

**Published:** 2020-07-27

**Authors:** Jakob Biran, Michael Gliksberg, Ido Shirat, Amrutha Swaminathan, Talia Levitas-Djerbi, Lior Appelbaum, Gil Levkowitz

**Affiliations:** 1 Department of Poultry and Aquaculture, Agricultural Research Organization, 7528809 Rishon, Letziyon, Israel; 2 Department of Molecular Cell Biology, Weizmann Institute of Science, PO Box 26, 7610001 Rehovot, Israel; 3 The Faculty of Life Sciences and the Multidisciplinary Brain Research Center, Bar-Ilan University, 5290002 Ramat-Gan, Israel

Correction to: *Scientific Reports* https://doi.org/10.1038/s41598-020-66447-2, published online 12 June 2020

In the original version of this Article, Supplementary Figures 1, 2 and 3 were uploaded as separate supplementary files. Furthermore, the Supplementary Figure Legends for Supplementary Figures 1, 2 and 3, as well as a Supplementary Table containing a list of oligonucleotides used for gRNA transcription and RT-PCRs were inadvertently omitted. These errors have been corrected in the Supplementary Information that now accompanies the Article.

