# Supplementary material for: Splice-specific deficiency of the PTSD-associated gene PAC1 leads to a paradoxical age-dependent stress behavior
Source: Sci Rep. 2020 Jun 12;10:9559. doi: 10.1038/s41598-020-66447-2 (PMC7292827; doi:10.1038/s41598-020-66447-2)
Supplement: Supplementary file 1 — Supplementary information. [file 41598_2020_66447_MOESM1_ESM.pdf]

Supplementary Information:

Splice-specific deficiency of the PTSD-associated gene PAC1 leads to a paradoxical age-dependent stress behavior

Jakob Biran[1*], Michael Gliksberg[2], Ido Shirat[2], Amrutha Swaminathan[2], Talia Levitas-Djerbi[3], Lior Appelbaum[3] and Gil Levkowitz[2*]

Department of Poultry and Aquaculture[1], Agricultural Research Organization, Rishon Letziyon 7528809, Israel.

Department of Molecular Cell Biology[2], Weizmann Institute of Science, PO Box 26, Rehovot 7610001, Israel

The Faculty of Life Sciences and the Multidisciplinary Brain Research Center[3], Bar-Ilan University, Ramat-Gan 5290002, Israel

*Correspondence should be addressed to

J.B. jakob@volcani.agri.gov.il

G.L. gil.levkowitz@weizmann.ac.il

Running title: Stress modulation by PAC1 splice isoform

Key words: adcyap1r1, gpcr, adcyap1, hypothalamus, stress, post-traumatic stress disorder, alternative splicing

Supplementary Figures

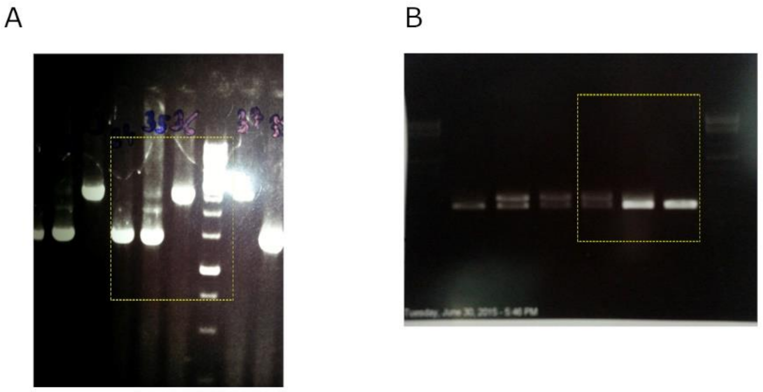

Supplementary Figure 1. Full length electrophoresis gel images of genomic DNA (**A**) and cDNA (**B**) in Figure 1. Images were cropped to the yellow dashed rectangle and color inverted.

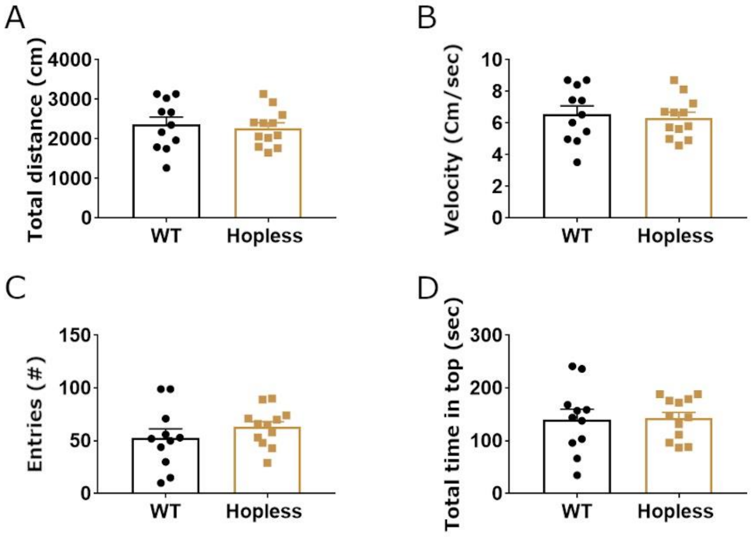

Supplementary Figure 2. Naïve *hopless* adults display normal behavior in novel tank diving assay. The behavioral response of adult *hopless* mutants (n=12) and WT siblings (n=11) to a novel tank was measured . The two genotypes exhibited a similar activity level, as seen in their total swimming distance swum and average swimming velocity during the 10 min test (**A** and **B**). Both genotypes displayed similar exploration of the top half of the tank (**C** and **D**). Data was analyzed using students-t test.

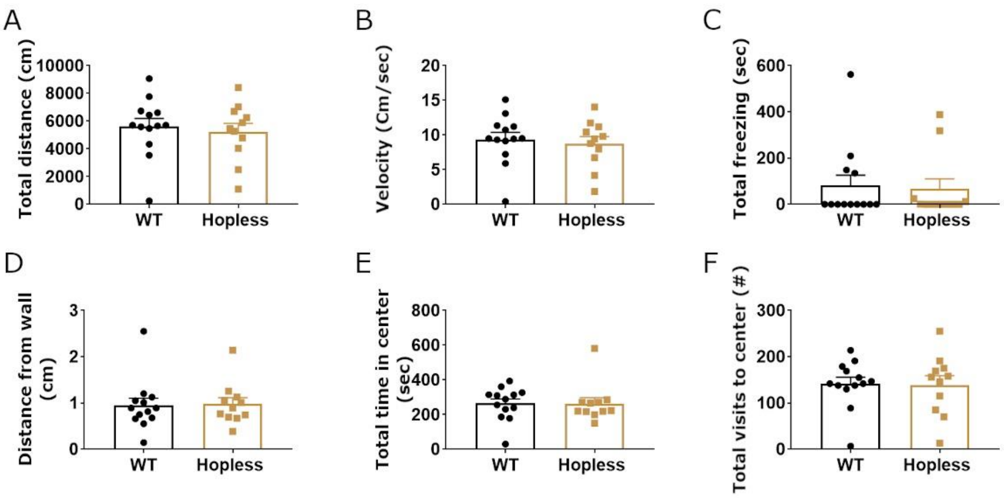

Supplementary Figure 3. Naïve adult *hopless* mutants display normal behavior in the open field assay. The behavioral response of adult *hopless* mutants (n=11) and WT siblings (n=13) in a novel open field arena was measured. Similarly to the novel tank diving assay, the two genotypes exhibited a similar activity level, as seen in their mean swimming distance and velocity, which significantly increased with time (**A** and **B**). In addition, both genotypes displayed significantly decreased freezing, distance from walls and time spent in the center of the arena along the test (**C**-**E**) and a slight but significant increase in their visits to the center (**F**), suggesting increased exploration activity over time. Data were analyzed using Student's *t*-test.

Supplementary Table 1. Oligonucleotides used for gRNA transcription and RT-PCRs

Primer	Sequence	Length (nt)
Hop genotyping 1F	CAAAGGTTGTTGGCGTCATA	20
Hop genotyping 2697R	ATTCCCCACGTTTGTCTCAG	20
Upstream hop gDNA	ATTTAGGTGACACTATAGTGTGAAAAGGAACAGTGTGTGTTTTAGAGCTAGAAATAGCAAG	60
Downstream hop gDNA	ATTTAGGTGACACTATATGGTGTGGACATTAAAGGAGGTTTTAGAGCTAGAAATAGCAAG	60
Constant gDNA	AAAAGCACCGACTCGGTGCCACTTTTTCAAGTTGATAACGGACTAGCCTTATTTTAACTTGCTATTTCTAGCTCTAAAAC	80
PAC1 cDNA – F	TGATAACACTGCCCTCTGGTG	21
PAC1 cDNA –R	CCAGGCCGAGCTCAAAGACC	20
